# Identifying contextual determinants of problems in tuberculosis care provision in South Africa: a theory-generating case study

**DOI:** 10.1186/s40249-021-00840-5

**Published:** 2021-05-10

**Authors:** Jamie Murdoch, Robyn Curran, André J. van Rensburg, Ajibola Awotiwon, Audry Dube, Max Bachmann, Inge Petersen, Lara Fairall

**Affiliations:** 1 School of Health Sciences, University of East Anglia, Norwich, NR4 7TJ UK; 2 University of Cape Town Lung Institute, Knowledge Translation Unit, University of Cape Town, Mowbray, 7700 South Africa; 3 Centre for Rural Health, University of KwaZulu Natal, Durban, South Africa; 4 Norwich Medical School, University of East Anglia, Norwich, NR4 7TJ UK; 5 King’s Global Health Institute, King’s College London, London, SE1 9NH UK; 6 Knowledge Translation Unit, Department of Medicine, University of Cape Town Lung Institute, University of Cape Town, Mowbray, 7700 South Africa

**Keywords:** Tuberculosis, Health systems strengthening, Primary healthcare, Person-centred care, Context

## Abstract

**Background:**

Despite progress towards End TB Strategy targets for reducing tuberculosis (TB) incidence and deaths by 2035, South Africa remains among the top ten high-burden tuberculosis countries globally. A large challenge lies in how policies to improve detection, diagnosis and treatment completion interact with social and structural drivers of TB. Detailed understanding and theoretical development of the contextual determinants of problems in TB care is required for developing effective interventions. This article reports findings from the pre-implementation phase of a study of TB care in South Africa, contributing to He**A**lth **S**ystem **S**tr**E**ng**T**hening in Sub-Saharan Africa (ASSET)—a five-year research programme developing and evaluating health system strengthening interventions in sub-Saharan Africa. The study aimed to develop hypothetical propositions regarding contextual determinants of problems in TB care to inform intervention development to reduce TB deaths and incidence whilst ensuring the delivery of quality integrated, person-centred care.

**Methods:**

Theory-building case study design using the Context and Implementation of Complex Interventions (CICI) framework to identify contextual determinants of problems in TB care. Between February and November 2019, we used mixed methods in six public-sector primary healthcare facilities and one public-sector hospital serving impoverished urban and rural communities in the Amajuba District of KwaZulu-Natal Province, South Africa. Qualitative data included stakeholder interviews, observations and documentary analysis. Quantitative data included routine data on sputum testing and TB deaths. Data were inductively analysed and mapped onto the seven CICI contextual domains.

**Results:**

Delayed diagnosis was caused by interactions between fragmented healthcare provision; limited resources; verticalised care; poor TB screening, sputum collection and record-keeping. One nurse responsible for TB care, with limited integration of TB with other conditions, and policy focused on treatment adherence contributed to staff stress and limited consideration of patients’ psychosocial needs. Patients were lost to follow up due to discontinuity of information, poverty, employment restrictions and limited support for treatment side-effects. Infection control measures appeared to be compromised by efforts to integrate care.

**Conclusions:**

Delayed diagnosis, limited psychosocial support for patients and staff, patients lost to follow-up and inadequate infection control are caused by an interaction between multiple interacting contextual determinants. TB policy needs to resolve tensions between treating TB as epidemic and individually-experienced social problem, supporting interventions which strengthen case detection, infection control and treatment, and also promote person-centred support for healthcare professionals and patients.

**Graphic abstract:**

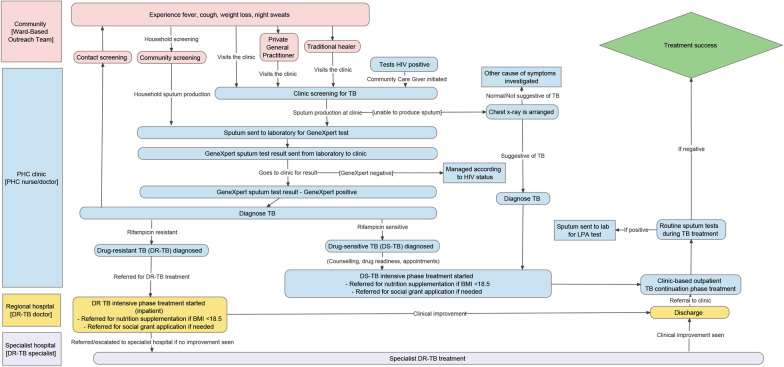

**Supplementary Information:**

The online version contains supplementary material available at 10.1186/s40249-021-00840-5.

## Background

Despite substantial progress and renewed political commitment to its eradication, 1.4 million deaths during 2019 makes tuberculosis (TB) the highest source of global infectious disease mortality before the coronavirus disease 2019 (COVID-19) pandemic [1]. The End TB Strategy established a key set of targets during the 2014 World Health Assembly, committing countries to achieve a 95% reduction in TB deaths and a 90% reduction in TB incidence (compared with 2015 levels) by 2035 [2]. While South Africa, long a high-burden TB country, has made important strides in this direction, the country’s TB epidemic continues to impose a substantial burden on its health system and people—South Africa’s 2019 TB incidence rate of 615 per 100 000 population is exceptionally high compared against the global average of 130 per 100 000 [1]; there is a 58% undetected TB burden in the community [3]; and in 2017 TB was the leading cause of death overall, contributing 6% of all deaths [4]. In addition to being among the top ten high-burden TB countries, South Africa has been designated one of the 20 countries where there is an overlap in high burdens of TB, multi-drug resistant tuberculosis (MDR-TB) and TB-HIV coinfection, with 58% prevalence of HIV among TB incident cases [1]. In 2018, the annual incidence of MDR-TB was 19 per 100 000, and the 71% treatment success for new and relapse TB cases in 2018 was substantially lower than the global average of 85%, partly due to the high case fatality rate [1]. MDR-TB poses a disproportionate burden on the South African TB budget (11% of total) because of the cost of medications used to treat MDR, including bedaqualine which South Africa has chosen to rollout widely [1]. These figures are especially troubling given that approximately 160 000 people with known active TB are lost to follow-up [4].

There are many reasons for the country’s protracted battle with TB. South Africa’s large socioeconomic inequities are well-known, and together with HIV, have led to TB flourishing among vulnerable populations who live in poverty, in high-density areas. Importantly, TB cannot be divorced from the country’s colonial and apartheid past, where labour migration from Bantustans to economic areas, was accompanied by little to no health system development for specific racial groups [5, 6]. Post-1994, many structural inequalities persisted, rapid urbanisation and the development of high-density living areas unfolded, along with abject poverty and further labour migration—especially in mining and industrial factory sectors [7]. The HIV epidemic in South Africa has been the most important determinant of TB incidence since 2000, with annual TB incidence rates increasing from around 400 per 100 000 population in 2000 to around 1300 per 100 000 population in 2007, as HIV prevalence increased [8]. Subsequently, the wide-scale implementation of antiretroviral treatment, which began in 2004 and reached 5.9 million people living with HIV by 2019, has been the main determinant of reductions in TB incidence to around 600 per 100 000 per year by 2019. A major achievement of the post-apartheid government was however the massive expansion of primary health care facilities, particularly in impoverished rural and urban areas, which had been severely lacking under apartheid. Although this provided reasonable geographical access to a network of about 3500 public sector primary care facilities throughout the country, providing free care, the quality of health care in these facilities was often lacking, partly due to constraints in the availability of adequately trained health professionals.

South Africa’s public health system underperforms in the implementation of national TB management guidelines, compared to targets. In 2018–2019, provincial TB screening rates (patients asked about TB symptoms) for patients aged 5 years and above ranged from 47 to 98%, with only two of the nine provinces reaching the 90% target. TB sputum testing for symptomatic patients over 5 years ranged from 36 to 99%, with just over half of all districts meeting the 90% target [4]. Failure to achieve the targets along the TB care cascade (screening, testing, diagnosis, linkage to care, retention to care) is associated with multiple health system delivery failures [9]. These include poorly organised and understaffed primary healthcare facilities, poor communication of test results, negative staff attitudes towards TB [10], poor integration of HIV and TB services, medication stock-outs, and inadequate information technology, infrastructure, data capture and monitoring systems [11]. The COVID-19 pandemic has added to the country’s TB health system woes. The focus on COVID-19 was accompanied by a rerouting of resources from TB programmes to address COVID-19, with dramatic reductions in TB case detection and treatment completion, leading some to warn that we risked greater losses from TB than from COVID-19 itself [1, 12]. Further, COVID-19 shares many symptoms with TB, increasing complexity of diagnosis, has competed for scarce mask and GeneXpert resources, and is associated with a more than two-fold risk of death especially during active disease [13].

In addition to these health system problems, the role of people and the patient as active components of the health system have been neglected, with patient level factors found to impact negatively on care-seeking and adherence. These include low levels of TB knowledge among service users [14], lack of patient empowerment [15], high levels of psychosocial distress that inhibit volition to self-manage [14]; dual stigma with HIV [10], and socio-economic disadvantage that impede people attending clinic appointments due to high transport costs and potential loss of income [16, 17].

Against this backdrop, the need for health systems strengthening that includes a person-centred orientation is essential. Person-centredness in care aims to: (1) give primacy to a person’s unique subjective experiences and interpretations of illness or disability by considering psychosocial dimensions alongside biomedical symptomology, (2) promote service-user empowerment in decision-making, and (3) prioritise relationships in care and treatment [18]. Our recent scoping review found little evidence of person-centred care interventions being deployed for people with tuberculosis [19]. Moreover, despite extensive documentation of TB programme failures, there has been little theoretical development of how contextual features of healthcare systems interact with people’s lives to produce poor service delivery and poor outcomes for patients. To address this gap, we conducted a case study to explore these relationships and generate theoretical propositions to inform development of a health system strengthening intervention to improve TB care delivery.

This study forms part of a broader five-year research programme (ASSET) with pre-implementation, intervention development, and pilot and evaluation phases, closely aligned with the Sustainable Development Goal of Universal Health Coverage, conducted across four countries—Ethiopia, Sierra Leone, South Africa, and Zimbabwe [20]. ASSET has an overall aim of developing and evaluating effective and sustainable health system strengthening interventions that support the translation of evidence-based practices that promote equitable person-centred care into routine health services. This article reports findings from the pre-implementation phase of one of the ASSET work packages to provide the theoretical basis for developing health systems strengthening interventions to strengthen person-centred TB care so as to improve key TB outcomes in South Africa.

## Methods

The study aimed to develop hypothetical propositions regarding the contextual determinants of problems in TB care in South Africa. To do so we devised a theory-building case study design [21] using mixed methods, comprising stakeholder interviews, observations of TB care, documentary review of national TB guidelines and policies and routinely available data collected between February and November 2019. To ensure coherence and wider theoretical generalisablity of findings across pre-implementation, intervention development and evaluation phases, we adopted the Context and Implementation of Complex Interventions (CICI) framework [22]. CICI is a determinant and evaluation framework comprising three dimensions—context, implementation and setting—which interact with one another and with the intervention. For the pre-implementation phase we focused on CICI’s seven contextual domains as a means for developing hypothetical propositions on the contextual determinants of problems in the delivery of TB care, including: geographical, epidemiological, socio-cultural, socio-economic, ethical, legal and the political domain.

### Study setting

The setting was six public-sector primary healthcare (PHC) facilities or clinics and one public sector hospital serving impoverished urban and rural communities in Amajuba district of KwaZulu-Natal province, South Africa (see Table 1). In 2017, 7.4% of all deaths in KwaZulu-Natal were due to TB [23], with a high case fatality rate of 11% [24], and in Amajuba TB represented the highest cause of death [23]. Amajuba district municipality is a geographically small (7102 km^2^) district in North-Eastern KwaZulu-Natal, comprised of three local municipalities (Newcastle, eMadlangeni and Dannhauser), eight towns, with a mix of urban, peri-urban and rural areas. The population is mainly isiZulu-speaking. The District has a total population of 556 580 (0.9% of South Africa) and 127 000 households, 12.3% of which live in informal dwellings. In 2019, 416 000 people lived in poverty, an increase of 11.3% from 2009. The largest economic sectors are community services, manufacturing, and financing [25].

**Table 1:** Characteristics of participating facilities

Site	Urban/Rural	Number of staff	Staff responsible for screening TB	Location of Sputum collection	TB care person	Monthly Average Number of patients*	Monthly Average TB screening
PHC Facility 1^a^	Rural	21	EN (Vital signs room)	Outside Area	1 PN	4279	Not available
PHC Facility 2 ^a, b^	Rural	38 (17 CCGs)		Outside area	1 PN	4941	5000
PHC Facility 3^a, b^^,^*	Urban	26	EN/ENA/PN (7 screening points)	Outside area	1 PN	8377	Not available
PHC Facility 4^b^	Rural	20 (10 CCGs)	EN (Vital signs room)	Outside area	1 PN	4492	2000
PHC Facility 5^b^	Rural	18 (25 CCGs)	EN (Vital signs room)	Outside area	1 PN	5387	4000
PHC Facility 6^b^	Rural	20 (17 CCGs)	EN (Vital signs room)	Outside area	1 PN	4826	2000
Hospital TB Outpatient 7^a^,*	Rural	16	Referral only	Outside area. Diagnostic sputum results received from PHC facilities	All staff	1772	Not available

*PN *professional nurse, *EN* enrolled nurse, *ENA* enrolled nurse assistant, *CCG* community caregiver, *PHC* primary healthcare,* TB* tuberculosis

^a^Facilities and hospital included in first stage of data collection

^b^Facilities included in second stage of data collection

* January 2018 to January 2019

TB care provision in Amajuba is governed by National TB guidelines and protocols (see Additional file 1 for description) [26, 27] and in 2019 the Amajuba district health department highlighted the need to address TB mortality as a key priority. Three PHC facilities and the hospital outpatient department were initially selected for inclusion and data collection was conducted in February 2019. We completed additional data collection in November 2019 in two of these PHC facilities, as well as an additional three facilities, at the request of the health department to assess screening processes and practices in greater detail.

### Study population, sampling and recruitment

#### Primary healthcare facility staff

We recruited facility managers to inform our understanding of the organisation of TB care; and purposively sampled nurses, doctors, counsellors and community caregivers who were treating patients at each of the primary care facilities in the selected district to be interviewed and/or observed. We also recruited nurses who were not routinely seeing TB patients as well as a private sector general practitioner and one traditional healer to understand their perspectives about the organisation of TB care, and of TB and the management of it.

#### Patients

To be eligible for interview, patients diagnosed with TB needed to have taken treatment for at least one month, in order to be able to inform us about their experience of care. Eligible patients who arrived at the facility on each day of data collection were consecutively sampled. Nurses identified eligible patients and informed the research team who then approached the patient about participation in individual interviews.

In the second stage of data collection, patients who screened positive for TB symptoms (‘presumptive TB’) were also interviewed after vital signs’ assessment. Nurses informed patients about the research, and if the patient was willing, notified the researcher who approached the individual about participating in a short interview. Informed consent was taken and interviews were audio-recorded.

#### Other stakeholders

Other key stakeholders were identified and purposively sampled to obtain a broader range of perspectives of service delivery, including researchers working for the Desmond Tutu Tuberculosis Centre (https://blogs.sun.ac.za/dttc/), TB managers at district and provincial level, and members of TB Proof, a NGO which started representing health workers with occupational TB and which now represents the views of people living with and surviving TB more generally (http://www.tbproof.org/who-we-are/).

### Data collection

#### Individual interviews

Interviews (see Table 2) were semi-structured and carried out in the language most appropriate to each participant (isiZulu = 29; English = 19), audio recorded, translated and transcribed in English. Interviews in English were conducted by RC (clinician and social scientist), AvR (social scientist), JM (social scientist) and AD (social scientist). Interviews in isiZulu were conducted by AD, supported by two fieldworkers trained in qualitative interview methods who lived locally and were known to the participating clinics. AD worked with the fieldworkers to translate the interview topic guides, identifying how best to adapt questions to retain the intended meaning when asked in isiZulu. The interview team carefully considered who was best placed to carry out different interviews so that participants would feel comfortable to disclose their experience and perceptions of TB care provision but also potentially sensitive topics such as stigma and psychological distress. The local fieldworkers were critical in this regard, facilitating insights into patients’ experiences which were perhaps less likely to be disclosed if conducted by another team member. Following national guidance on the ethics of payment, incentives to participate were not offered as interviews took place following the patient’s consultation and did not require additional travel or time off work to be interviewed [28].

**Table 2:** Characteristics of interviewed participants

Health care workers		Facility Managers	5
		Provincial managers	1
		TB district managers	1
		Community Caregivers	2
		Doctors	3
		Professional Nurses	3
		Traditional Healer	1
Non-Governmental Organisations		Desmond Tutu TB centre researchers	1
		TB Proof members	2
Patients Diagnosed with TB	Gender	Male	13
		Female	7
	Time on treatment	More than 1 month, duration uncertain	2
		Less than 2 months	9
		2–8 months	6
		Post treatment	3
	Type of treatment	Retreatment	3
		First treatment	17
	Place of diagnosis	Hospital	11
		Clinic	5
		General Practitioner	4
	Type of TB	MDR-TB	6
		Drug sensitive TB	14
Patients with Presumptive TB		Male	5
		Female	4

*TB* tuberculosis,* MDR-TB* multi-drug resistant tuberculosis

For individual interviews with patients, semi structured questions were avoided to minimise the researcher imposing their own assumptions on participants’ experiences during the interview. Instead the researcher elicited stories [29] of patients’ journeys through the TB care pathway from the point where they first noticed symptoms through to treatment and followed up topics as they arose. Patients who screened positive for TB were briefly interviewed to elicit their understanding of the screening questions, instructions for providing sputum testing and next steps for their care. 

Interviews with nurses, counsellors, community caregivers and doctors explored the provision of TB care, implementation of infection control measures, and solutions for strengthening TB care. Interviews with provincial managers and stakeholders explored current services and interventions to support people with TB, interventions to reduce TB infection and address psychological needs of patients with TB, and perspectives on required interventions to improve service delivery at a system wide, organisational and individual facility level.

Interviews conducted in isiZulu were translated and transcribed by one of the fieldworkers to help ensure the meaning of participants’ responses were retained rather than a direct literal translation. RC and AD then reviewed all isiZulu recordings and transcripts to identify and resolve any potential misrepresentation from the original meaning.

#### Healthcare facility observations

Within all six primary healthcare facility we carried out periods of direct, non-participant observations within non-clinical areas to understand the organisation and process of TB care including patient flows, TB screening and testing, infection control measures and data capturing processes. We recorded contemporaneous written field notes of their observations using a semi-structured observation guide (Additional File 2).

#### Documentary and routine data review

Relevant policy and guidelines (Additional File 1), district TB mortality reports and routine data [24] were reviewed to identify best practices for TB screening, testing, diagnosis, treatment initiation, infection control and follow-up of patients, mortality burden and data system bottlenecks.

### Data analysis

**Fig. 1:**
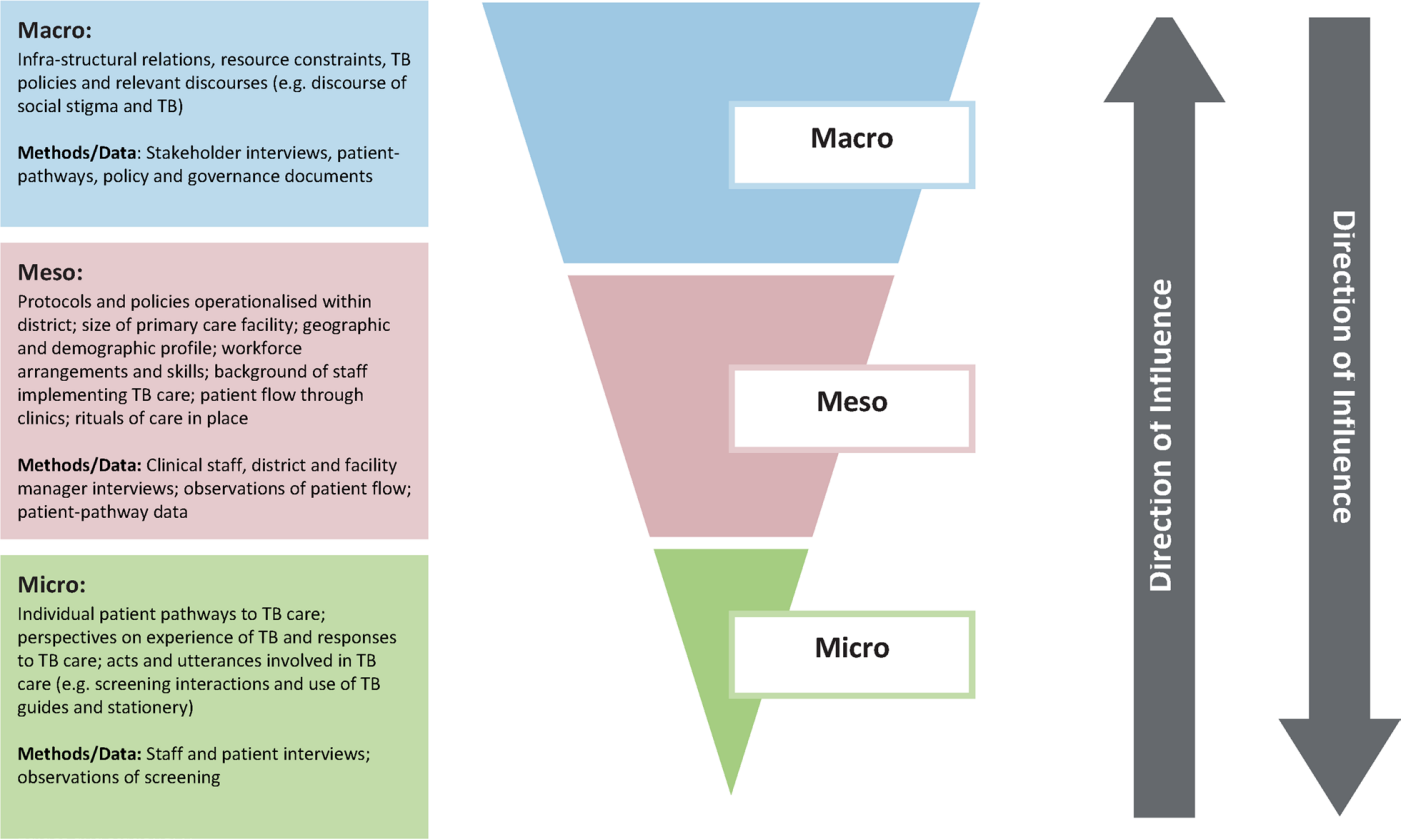
Contextual framework for investigating TB care in Amajuba district. *TB* tuberculosis

In order to generate hypothetical propositions on the contextual determinants of problems in TB care delivery, we identified relationships between CICI domains and across macro-contextual features (e.g. national and international healthcare policy, discourses, infra-structural relations, socio-economic factors), meso-contextual features (i.e. organisation of TB care at a primary healthcare facility level), and micro-contextual features (i.e. patients’ and clinician’s behaviour). We drew on Braun and Clarke’s thematic analysis as a ‘contextualist’ method [30], examining how macro-contextual features shaped meso and micro (or vice versa), thereby tracing a thread between specific perspectives or observations to the broader social historical context in which they were manifested (see Fig. 1). Rather than necessarily developing higher-order themes within the discrete datasets, this approach required treating each participant report or observation as a potential contextual feature which we then explored within and across contextual levels and across data types to develop and test emerging theories [31], for example how reported implementation of infection control measures matched recommended practice within TB guidelines as well as our observations within the facility. This iterative approach enabled us to transition from the particularities of Amajuba as a single case to theoretical explanations of how different contextual determinants applicable in other South Africa settings may shape the patterns we observed, facilitating generalisable inferences and predictions on what kinds of intervention components are needed to tackle different contextual determinants of problems in TB care.

All interviews were inductively coded for features using the qualitative data analysis software NVivo 12 (QSR International, https://www.qsrinternational.com/nvivo/home). This provided detailed staff, manager and stakeholder perspectives of the process and content of TB care in facilities; and for patients, pathways to care, and experiences of living with and managing TB. Initially four researchers (RC, AD, JM, AvR) coded two of the same interviews and compared these to identify and resolve differences. The facilities were then divided between these research members, with the coding of features checked by JM. A constant comparison approach was adopted, working iteratively between data obtained from different interviewees within and between facilities to test out categories, including searching for disconfirming cases [32]. First order codes were then analysed to consider if they could be developed into higher order codes to better facilitate understanding of emerging relationships between contextual features. Field notes were analysed to provide a detailed description of the process and content involved in provision of TB care, including screening, testing, data capture and infection control measures.

#### Data synthesis within CICI framework

As the analysis developed we mapped contextual features onto the seven contextual domains of the CICI Framework. Any feature which did not readily map onto a domain was discussed and assigned to a domain or an additional domain added. We then analysed the mapped domains in light of emerging theories to generate hypothetical propositions which specified the contextual determinants of problems in delivering effective person-centred TB care. Finally, we hypothesised which intervention components and implementation strategies would logically tackle those determinants. Throughout analysis, we held regular meetings between all project team members and the district health department to review findings, discuss emerging theories on the relationship between features and contextual domains, and later develop hypothetical propositions and intervention components.

### Ethical considerations

The key ethical principles of voluntary and informed participation, confidentiality and safety of participants were used in all researcher and participant interactions. Written consent for interviews was obtained from all stakeholders, facility managers, clinicians and patients. Facility managers provided consent for observations of non-clinical areas. All participants were provided with written information about the research, informed that their participation was voluntary and that they could withdraw from participation at any time. Patients were typically approached after their consultation and interviews were conducted in outdoor areas with researchers wearing a mask where possible.

## Results

We identified a diversity of contextual features of TB care which cut across the CICI framework’s epidemiological, socio-economic, political, socio-cultural, ethical, legal and geographical domains, and an additional institutional domain (Additional file 3). Contextual determinants of problems in TB care interacted across macro-, meso- and micro-contextual levels contributing to delayed TB diagnosis, limited support for staff and patients’ psychosocial needs, patients lost to follow-up after diagnosis, and inadequate infection control within PHC facilities (Additional file 4).

### Delayed diagnosis at community and facility level

**Fig. 2:**
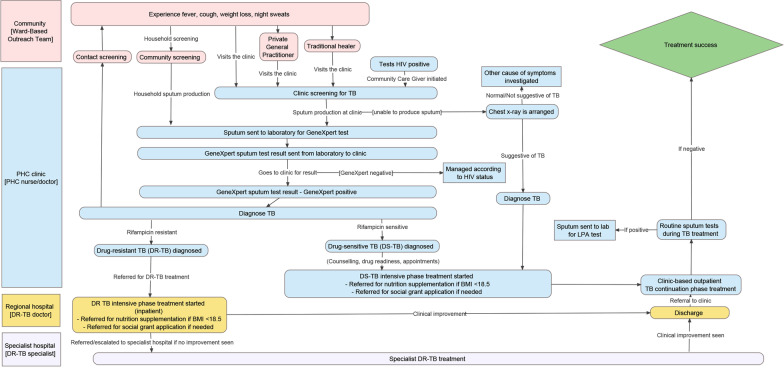
Process Map of TB Care in Amajuba. *DR-TB* drug resistant tuberculosis, *TB* tuberculosis, *DS-TB* drug-sensitive tuberculosis, *PHC* primary healthcare

Patients reported a range of referral pathways (Fig. 2) from the point of noticing symptoms to diagnosis and treatment initiation, in one case involving three doctors, (including private doctors) over a two-month period before being diagnosed with MDR-TB. Fragmented data recording and sharing underpinned such delays which was also reflected in delays retrieving sputum test results from laboratories, leading to loss of follow up or delayed diagnosis and treatment. Within facilities, there was typically one room dedicated for TB patients and care provided by one professional nurse. Ideal Clinic policy requires that nurses screen patients for TB in vital signs rooms, asking four questions: ‘Do you have a cough?’; ‘Do you have a fever?’; ‘Have you lost weight?’; and ‘Are you sweating a lot at night?’ However, we observed wide variation in TB screening practices, taking place within and outside vital signs rooms and inconsistent questioning of patients, sometimes only occurring if patients showed signs or symptoms. Within some facilities, data recording of patients screened was incomplete and inaccurate, including discrepancies between numbers of patients observed and recorded; and missing screening data for chronic condition patients. Instructions to provide sputum at home or in the clinic varied both within and between clinics, with some patients asked to revisit the clinic with the sample. However, patients screening positive for TB were not recorded in a TB register until they returned their sputum. Patients also reported being confused about how to provide sputum, how much sputum to produce and when and where to return the sample. Only two facilities reported that they supervised sputum collection. This was reflected in high rejection rates of sputum samples. For the period of January to August 2019, the two main reasons for rejected samples in the district were insufficient specimen (44%) and leaked specimens (30%).

Other issues causing delays were patients having a preference for private doctors, accessing traditional healers as a first point of contact and delayed presentation to public sector health services. Several staff reported that patients provided incorrect addresses citing privacy concerns including not wanting to be seen attending their local clinic or having CCGs follow them up in their homes.

### Limited support for psychosocial needs of staff and patients

Patients reported how key aspects of their life had been lost since being diagnosed with TB, including control of their body, mental state, professional role, social contact and personal relationships. These different forms of loss can be seen to represent a ‘loss of self’ [33] as patients struggled to adjust to the changes in their lives, highlighting a need to provide psychosocial support from the point of diagnosis through to treatment completion. However, both staff and patients frequently presented care as having a biomedical focus on treatment adherence, with limited discussion of the psychosocial support patients may require or how to continue with everyday activities that are important to patients.

The loss of social contact and personal relationships arising from patients choosing to self-isolate during the early weeks of their treatment was intimately connected to a moral incentive not to infect others. For some this created a stigmatising element to their relationships, with friends or relatives distancing themselves from the diagnosed patient. Stigmatisation and fear of being infected shaped how some staff reported being stressed about providing TB care, and in one facility we observed staff visibly recoiling at the suggestion that they might need to take on this role. An exception to this was one nurse whose expressed commitment to caring for individuals living with TB functioned to articulate the otherwise wider stigmatisation and marginalisation of this group: ‘*You know if people are sick with TB, nobody loves them. Nobody likes them. Nobody wants anything to do with them. I said, if they cannot be loved by anyone, why can’t I love them. That is what keeps me here each and every day. Just to give my love to them. To understand them and to love them, and to attend to their needs.’* (TB nurse).

### Patients lost to follow-up after diagnosis or end of treatment initiation phase

An important contextual feature shaping the ability of patients to adhere to treatment and for facilities in following patients through to treatment completion is the level of unemployment, at 39.1% in 2014 [34]. A large proportion of the community live in high-density housing, which is likely to increase the risk of TB transmission. Patients and clinicians reported difficulties in attending facilities to collect treatment (and therefore medication adherence) because transport costs are required. Many patients are daily paid workers within manufacturing industries with working conditions which restrict their ability to flexibly attend clinic appointments. Related to this are working migration patterns, with some patients working away from home and then returning to collect medication, potentially creating discontinuities in treatment, made worse if patients provide the wrong address or move without informing the facility.

The reported focus of consultations on ensuring patients were adherent to medication was set against little evidence of patients being supported to deal with medication side-effects either through expressions of empathy or provision of medication to assist with them.

### Inadequate infection control alongside policy to minimise TB stigma

A particular challenge for minimising the stigma surrounding TB is the subtle interaction with the need to take appropriate infection control measures. Integration of HIV care into chronic care services has helped reduce stigma in South African primary care [35, 36]. Ideal Clinic policy builds on this by extending this arrangement to those with TB, after the intensive phase of treatment has been completed. While these patients should be non-infectious, in practice we observed patients with TB at various stages of treatment sitting with others attending for other chronic conditions. We also noted inconsistent use of masks by nurses and patients, potentially revealing spaces where the tension between infection control and stigma creates individual uncertainty about what measures are appropriate and should be taken to reduce infection.

Primary care facility infrastructure did not help minimise the spread of TB, including long corridors with few windows. We observed patients with TB sat close by other patients including pregnant women and children, and clinical staff reported the difficulties in maintaining appropriate infection control measures. This included patients already commencing TB treatment and those yet to start treatment, with the latter posing a greater risk of transmission than the former. In contrast, at the hospital site, dedicated to treating both drug-sensitive and MDR-TB, patients sat in open air waiting areas, masks were routinely worn in all inpatient wards and outpatient consultation rooms, and a team of staff deployed to treat the range of patients requiring care.

## Discussion

Achieving WHO End TB strategy targets [2] urgently requires interventions that strengthen the healthcare system to better identify, test and diagnose people with TB and to support them to successfully complete the TB treatment pathway. Our findings highlight that to do so requires careful consideration of how different contextual features interact to produce different problems in the delivery of TB care. We have provided hypothetical propositions, and recommendations for potential intervention components and implementation strategies to facilitate this process, pinpointing relationships between policy, the infrastructural organisation of primary healthcare, and discourse at a macro-contextual level; the operationalisation of policy within primary care at a meso-level; and how these contextual conditions come to be manifested at a micro-level where patients seek and receive care (see Additional file 4).

There is a pressing need to improve systems of TB screening, testing, data capture as well as developing interventions that function to destigmatise TB within communities. Patients frequently present to services some time after onset of symptoms and may have multiple contacts over weeks or months before receiving a diagnosis. Fragmented service provision and under-resourced public services reinforce these delays undermining data sharing, referrals, and efficient screening and testing procedures. Within clinics, we identified variable quality in TB screening, sputum testing and data capture, and patients were unclear about how to provide sputa samples. Strengthening patient education and systems for screening and testing within public facilities are required to reduce delays to diagnosis and treatment, supporting similar findings in previous research [9–11]. Although some determinants such as poverty and distribution of employment are societal challenges that require systemic intervention over decades, recognition of these difficulties highlights the need to ensure patients taking TB medication have access to food [15], as well as the potential of inter-sectoral collaboration between health and industry to support employees to attend facilities in different locations [37].

Our study identified specific tensions between two broad policy agendas that have very different underpinning assumptions and framings of what type of problem TB represents, shaping what practices healthcare professionals enact and therefore the care of patients. On the one hand, TB policy [1, 2] is predominantly orientated towards reducing the mortality and incidence of TB, which discursively frames the illness as a *population-based disease epidemic*, inherently placing TB screening, testing, diagnosis, treatment adherence and TB infection control measures at the heart of protocols and practice for healthcare facilities, healthcare workers, patients and communities. At the same time, there is recognition that this agenda is at odds with a person-centred view of TB care [2], which constructs the condition as an *individually-experienced social problem*, facilitating understanding of how TB has impacted on individual’s participation in society and their associated psychological, emotional and mental health.

These different orientations are not necessarily mutually exclusive. However, our findings highlight how the global discourse of TB as epidemic can dominate clinical practice at the expense of the individual, seen in terms of patient’s psychosocial problems not being elicited within consultations, but also the individual concerns of healthcare workers who may be the sole person responsible for TB care. The realities of operationalising these two agendas without one undermining the other requires multi-faceted and nuanced strategies for supporting healthcare workers and patients through the TB treatment pathway. Disease-specific policies and guidelines in low and middle-income countries (LMICs) have historically functioned to verticalise care of chronic illnesses [38, 39], and despite the promotion of integrated care within facilities, this institutionalised practice places responsibility for meeting TB targets in the hands of individual nurses. South Africa’s Integrated Chronic Disease Management programme has also made progress in integrating primary care for HIV and NCDs, but TB care remains separate, partly due to the need to prevent transmission within facilities and report on TB treatment outcomes. Important interventions therefore lie in ensuring TB policy recognises the challenges clinicians face in providing TB care, identifying strategies for enabling the collective management of TB, training all staff to provide that care, and providing support for staff to manage anxieties about TB infection.

Such interventions broaden the scope of person-centred care to incorporate clinicians in addition to patients and their families, and in doing so, may function to facilitate more holistic care of individual patients. As well as integrating care for HIV, TB and non-communicable conditions, prioritising a focus on the relationship between psychosocial health and TB treatment adherence may be critical for supporting the emotional, social and mental health needs of patients as a mechanism to enhance treatment adherence. Evidence is already available which has demonstrated the links between stigma, depression, family and community support, and non-adherence for patients with TB [40–46]. However, efforts to improve TB treatment outcomes have not prioritised identification and management of stigma and depression, and research in this area remains scarce and limited to MDR-TB or those hospitalised for treatment. Our recent scoping review [19] which identified a paucity of person-centric TB care being delivered across LMICs, found only two studies, both based in Nepal, which focused on psychosocial support of patients with MDR-TB [47, 48].

Interventions that orientate to the interaction between TB stigma and psychosocial health might be undermined by infection minimisation strategies that function to reinforce and perpetuate such stigma. South Africa introduced a TB infection control programme in 2007 (updated in 2012) with a structural review of healthcare facilities and TB infection control practices, as well as development of minimum standards for health facilities [49]. However, while in most high-income countries it is standard practice to isolate people who are potentially infected with TB, in many LMICs this rarely happens and is often perceived to be discriminatory [47, 50]. The boundaries between appropriate infection control measures and actions that function to reinforce TB stigma are therefore blurred and despite the availability of legislation, guidelines and policies, we found implementation of TB infection control is generally poor and adherence of health care workers is sub-optimal, echoing previous research [51–53]. This has created an ethical paradox between attempts to minimise TB stigma and the implementation of infection control policies which have arguably led to indecision within healthcare facilities about what infection control measures are appropriate and consequentially the less than optimal practices we observed.

The range of factors that perpetuate the South African TB epidemic have been rendered all the more complex with the onset of the COVID-19 pandemic, which has already had dire consequences for TB care [54]. Not only is little known about the incidence, risk and course of illness of COVID-19 in people with undiagnosed pulmonary, drug-resistant or complex TB presentations but COVID-19 shares many symptoms with TB, such as cough, fever, and shortness of breath, making differentiating between COVID-19 and TB challenging for healthcare workers. On the other hand, the normalisation of mask wearing and increased awareness around infection control practices has potential to destigmatise these measures for TB care in the longer term.

This study took place in one district in South Africa with a high burden of TB, limiting direct transferability of our findings to other parts of South Africa and other LMICs. However, by focusing on Amajuba as a theory-building case study [21] we have generated hypothetical propositions which trace a thread between global forces on the identification, diagnosis and treatment of TB and the actions of individual clinicians and patients who face the realities of managing TB in everyday life. In doing so we offer theoretical generalisability on the contextual determinants of TB that echo across LMICs with a high burden of TB, providing a foundation for developing interventions to tackle those determinants.

In setting out the domains and contextual determinants of TB care, we are conscious that the foundations of our findings lie in the situated perspectives of participants and our own observations of TB care. We are not claiming to have objectively established causal relationships between domains, TB care and rates of TB cases and fatalities. However, the breadth of data we have collected, the depth of analysis and triangulation across data types provide strong foundations for the hypothetical propositions we propose. Concrete intervention development work is now required, using the recommendations for intervention components and implementation strategies as a starting point for further development and specificity.

Our sample was limited by staff availability and according to which patients presented on the day of data collection and whom were willing to participate. Negotiating access to facilities to conduct fieldwork required numerous communications to build trust between district managers, facility staff and the research team, which in itself revealed the burden of accountability experienced by staff responsible for reducing TB morbidity and mortality within this district. Interviews and observations were sometimes difficult to conduct in busy facility environments which limited our ability to purposively select patients and screening assessments.

## Conclusions

Significant health systems strengthening interventions are required if WHO targets for TB are to be achieved in South Africa by 2035. To do so requires careful consideration of how different contextual determinants interact to produce problems in the delivery of TB care. Strengthening the quality and processes for screening, testing and diagnosing patients within primary healthcare facilities are essential but need to be supported by policy that resolves tensions between treating TB as a population-based epidemic and TB as an individually-experienced social problem. At the heart this are TB nurses who need to practise in a climate of stigma, infection control measures and accountability for ensuring patients complete the TB treatment pathway. Structuring TB care as a collective endeavour within facilities underpinned by a person-centred ethos of support for colleagues may help ensure the same principles are translated to supporting patients to manage not just the condition but their experience of TB. Future research should focus on co-producing health system strengthening interventions and implementation strategies with staff, policymakers and patients to help ensure the different priorities of these respective stakeholders can be aligned and feasibly delivered in everyday clinical practice. Such an approach will coherently follow from understanding the complex interaction between contextual determinants of TB care provision, including how COVID-19 contributes to that complexity. Subsequent mixed methods implementation research will therefore be critical, designed to provide robust evidence for wide-scale implementation of multi-faceted, health systems strengthening and person-centred interventions which reduce TB incidence and deaths.

## Supplementary information

**Additional file 1.** Provision of TB care in Amajuba district, South Africa.

**Additional file 2.** Guide for conducting observations of non-clinical areas.

**Additional file 3.** Contextual domains of tuberculosis care provision.

**Additional file 4.** Contextual determinants, hypothetical propositions and intervention components for tackling problems in TB care.

## Data Availability

The datasets generated and/or analysed during the current study are not publicly available due to data transcripts including personal participant information not suitable for sharing, but are available from the corresponding author on reasonable request.

## Abbreviations

AFB: Acid-fast bacilli
ART: Antiretroviral treatment
ARV: Antiretroviral
CCG: Community caregiver
CHC: Community Health Centre
CICI: Context and Implementation of Complex Interventions
DR-TB: Drug resistant tuberculosis
DS-TB: Drug-sensitive tuberculosis
DOTS: Directly observed treatment short course
EN: Enrolled nurse
ENA: Enrolled nurse assistant
LMIC: Low and middle-income countries
LPA: Line probe assays
MDR-TB: Multi-drug resistant tuberculosis
OPD: Outpatient Department
PHC: Primary healthcare
PN: Professional nurse
TB: Tuberculosis

## References

[1] World Health Organisation (2020). Global tuberculosis report 2020.

[2] World Health Organisation (2014). The End TB Strategy: Global strategy and targets for tuberculosis prevention, care and control after 2015.

[3] The Knowledge Hub. The First National TB Prevalence Survey - South Africa 2018. 2021. https://www.knowledgehub.org.za/elibrary/first-national-tb-prevalence-survey-south-africa-2018. Accessed 08 Mar 2021.

[4] Massyn N, Barron P, Day C, Ndlovu N, Padarath A (2020). District Health Barometer 2018/19.

[5] Packard RM (1989). White plague, black labor: Tuberculosis and the political economy of health and disease in South Africa.

[6] Farmer P (2003). Pathologies of power: health, human rights, and the new war on the Poor Berkeley.

[7] Churchyard GJ, Mametja LD, Mvusi L, Ndjek N, Hesseling AC, Reid A (2014). Tuberculosis control in South Africa: successes, challenges and recommendations. SAMJ..

[8] World Health Organisation. Tuberculosis profile: South Africa. 2021. https://worldhealthorg.shinyapps.io/tb_profiles/?_inputs_&entity_type=%22country%22&lan=%22EN%22&iso2=%22ZA%22. Accessed 08 Mar 2021.

[9] Naidoo P, Theron G, Rangaka MX, Chihota VN, Vaughan L, Brey ZO, et al. The South African Tuberculosis Care Cascade: Estimated Losses and Methodological Challenges. J Infect Dis. 2017;216(suppl_7):S702-S13. 10.1093/infdis/jix335 PMC5853316 29117342

[10] Skinner D, Claassens M (2016). It's complicated: why do tuberculosis patients not initiate or stay adherent to treatment? A qualitative study from South Africa. BMC Infect Dis..

[11] Loveday M, Padayatchi N, Voce A, Brust J, Wallengren K (2013). The treatment journey of a patient with multidrug-resistant tuberculosis in South Africa: is it patient-centred?. Int J Tuberc Lung Dis..

[12] Zhou S, Van Staden Q, Toska E (2020). Resource reprioritisation amid competing health risks for TB and COVID-19. Int J Tuberc Lung Dis..

[13] Boulle A, Davies M-A, Hussey H, Ismail M, Morden E, Vundle Z (2020). Risk factors for COVID-19 death in a population cohort study from the Western Cape Province South Africa. Clin Infect Dis..

[14] Theron G, Peter J, Zijenah L, Chanda D, Mangu C, Clowes P (2015). Psychological distress and its relationship with non-adherence to TB treatment: a multicentre study. BMC Infect Dis..

[15] Goudge J, Gilson L, Russell S, Gumede T, Mills A (2009). Affordability, availability and acceptability barriers to health care for the chronically ill: longitudinal case studies from South Africa. BMC Health Serv Res..

[16] Maswanganyi NV, Lebese RT, Mashau NS, Khoza LB (2014). Patient-perceived factors contributing to low tuberculosis cure rate at Greater Giyani healthcare facilities. Health SA Gesondheid..

[17] Birch S, Govender V, Fried J, Eyles J, Daries V, Moshabela M (2016). Does treatment collection and observation each day keep the patient away? An analysis of the determinants of adherence among patients with Tuberculosis in South Africa. Health Policy Plan..

[18] Wilberforce M, Challis D, Davies L, Kelly MP, Roberts C, Loynes N (2016). Person-centredness in the care of older adults: a systematic review of questionnaire-based scales and their measurement properties. BMC Geriatr..

[19] van Rensburg A, Dube A, Curran R, Ambaw F, Murdoch J, Bachmann M (2019). Comorbidities between tuberculosis and common mental disorders: a scoping review of epidemiological patterns and person-centred care interventions from low-to-middle income and BRICS countries. Infect Dis Poverty..

[20] Seward N. et al. HeAlth System StrEngThening in four sub_Saharan African countries (ASSET) to achieve high-quality, evidence-informed surgical, maternal and newborn, and primary care: protocol for pre-implementation phase studies. Health Res Policy Syst. (under review). 10.1080/16549716.2021.1987044 PMC8765245 35037844

[21] Thomas G (2011). A typology for the case study in social science following a review of definition, discourse, and structure. Qual Inquiry..

[22] Pfadenhauer LM, Gerhardus A, Mozygemba K, Lysdahl KB, Booth A, Hofmann B (2017). Making sense of complexity in context and implementation: the Context and Implementation of Complex Interventions (CICI) framework. Implement Sci..

[23] Statistics South Africa. Mortality and causes of death in South Africa: Findings from death notification. Pretoria; 2017.

[24] Hlongwa M, Ngozo J, Tshabala M (2020). Profiling TB deaths in Amajuba District.

[25] Affairs DoCGaT. Profile and Analysis: District Development Model 01/52. . Pretoria: CoGTA. 2020. https://www.cogta.gov.za/ddm/wp-content/uploads/2020/07/2020.07.04-Amajuba-District-Profile-Edited-Final.pdf. Accessed 08 Mar 2021.

[26] National Department of Health (2015). National Infection Prevention Control Guideline for TB.

[27] National Department of Health. National Tuberculosis Management Guidelines. Republic of South Africa; 2014.

[28] NHREC. Payment of trial participants in South Africa: Ethical considerations for Research Ethics Committees (RECs). NHREC; 2012.

[29] Briggs C (1986). Learning how to ask: a sociolinguistic appraisal of the role of the interview in social science research.

[30] Braun V, Clarke V (2006). Using thematic analysis in psychology. Qual Res Psychol..

[31] Burawoy M (1998). The extended case method. Sociol Theory.

[32] Miles MB, Huberman AM (1994). Qualitative data analysis, 2nd.

[33] Charmaz K (1983). Loss of self: a fundamental form of suffering in the chronically ill. Sociol Health Illn..

[34] Department of Health Province of KwaZulu-Natal. Amajuba District Health Plan 2018/19–2020/21. Department of Health; 2020.

[35] Mathibe MD, Hendricks SJ, Bergh AM (2015). Clinician perceptions and patient experiences of antiretroviral treatment integration in primary health care clinics Tshwane, South Africa. Curationis..

[36] Ameh S, Klipstein-Grobusch K, D'Ambruoso L, Kahn K, Tollman SM, Gomez-Olive FX (2017). Quality of integrated chronic disease care in rural South Africa: user and provider perspectives. Health Policy Plan..

[37] Hartel LA, Yazbeck AS, Osewe PL (2018). Responding to health system failure on tuberculosis in Southern Africa. Health Syst Reform..

[38] Bates M, Marais BJ, Zumla A (2015). Tuberculosis comorbidity with communicable and noncommunicable diseases. Cold Spring Harb Perspect Med..

[39] Marais BJ, Lonnroth K, Lawn SD, Migliori GB, Mwaba P, Glaziou P (2013). Tuberculosis comorbidity with communicable and non-communicable diseases: integrating health services and control efforts. Lancet Infect Dis..

[40] Qiu L, Tong Y, Lu Z, Gong Y, Yin X (2019). Depressive symptoms mediate the associations of stigma with medication adherence and quality of life in tuberculosis patients in China. Am J Trop Med Hyg..

[41] Munro SA, Lewin SA, Smith HJ, Engel ME, Fretheim A, Volmink J (2007). Patient adherence to tuberculosis treatment: a systematic review of qualitative research. PLoS Med..

[42] Chakrabartty A, Basu P, Ali KM, Ghosh D (2019). Tuberculosis related stigma attached to the adherence of Directly Observed Treatment Short Course (DOTS) in West Bengal. India. Indian J Tuberc..

[43] Kipp AM, Pungrassami P, Stewart PW, Chongsuvivatwong V, Strauss RP, Van Rie A (2011). Study of tuberculosis and AIDS stigma as barriers to tuberculosis treatment adherence using validated stigma scales. Int J Tuberc Lung Dis..

[44] Chowdhury MR, Rahman MS, Mondal MN, Sayem A, Billah B (2015). Social impact of stigma regarding tuberculosis hindering adherence to treatment: a cross sectional study involving tuberculosis patients in Rajshahi City Bangladesh. Jpn J Infect Dis..

[45] Ambaw F, Mayston R, Hanlon C, Medhin G, Alem A (2018). Untreated depression and tuberculosis treatment outcomes, quality of life and disability Ethiopia. Bull World Health Organ..

[46] Woith WM, Larson JL (2008). Delay in seeking treatment and adherence to tuberculosis medications in Russia: a survey of patients from two clinics. Int J Nurs Stud..

[47] Baral SC, Karki DK, Newell JN (2007). Causes of stigma and discrimination associated with tuberculosis in Nepal: a qualitative study. BMC Public Health..

[48] Khanal S, Elsey H, King R, Baral SC, Bhatta BR, Newell JN (2017). Development of a patient-centred, psychosocial support intervention for multi-drug-resistant tuberculosis (MDR-TB) care in Nepal. PLoS One..

[49] Republic of South Africa (2017). Let our actions count: South Africa's national strategic plan for HIV, TB and STIs: 2017–2022.

[50] Chang SH, Cataldo JK (2014). A systematic review of global cultural variations in knowledge, attitudes and health responses to tuberculosis stigma. Int J Tuberc Lung Dis..

[51] Farley JE, Tudor C, Mphahlele M, Franz K, Perrin NA, Dorman S (2012). A national infection control evaluation of drug-resistant tuberculosis hospitals in South Africa. Int J Tuberc Lung Dis..

[52] Kanjee Z, Amico KR, Li F, Mbolekwa K, Moll AP, Friedland GH (2012). Tuberculosis infection control in a high drug-resistance setting in rural South Africa: information, motivation, and behavioral skills. J Infect Public Health..

[53] Sissolak D, Bamford CM, Mehtar S (2010). The potential to transmit Mycobacterium tuberculosis at a South African tertiary teaching hospital. Int J Infect Dis..

[54] Boffa J, Mhlaba T, Sulis G, Moyo S, Sifumba Z, Pai M (2020). COVID-19 and tuberculosis in South Africa: a dangerous combination. S Afr Med J..

