# Supplementary material for: Identifying contextual determinants of problems in tuberculosis care provision in South Africa: a theory-generating case study
Source: Infect Dis Poverty. 2021 May 10;10:67. doi: 10.1186/s40249-021-00840-5 (PMC8108019; doi:10.1186/s40249-021-00840-5)
Supplement: Supplementary file 1 — Additional file 1. Provision of TB care in Amajuba district, South Africa. [file 40249_2021_840_MOESM1_ESM.docx]

**Provision of TB care in Amajuba district, South Africa**

Patients with symptoms are required to present to primary care for evaluation which should include testing for TB in those with a cough of more than two week’s duration, and of any duration if known to be HIV positive. Such passive case detection is augmented by routinely asking all attendees about four cardinal symptoms of TB (cough, weight loss, fever, night sweats). Screening at clinic level is supposed to take place in ‘vital signs’ rooms where patients’ folders are reviewed, and basic measurements like blood pressure performed, usually by Enrolled Nurses (ENs) or Enrolled Nursing Assistants (ENAs). Professional Nurses (PNs) review screening results in consulting rooms. Screening questions are also deployed by community care workers, in KwaZulu-Natal known as Community Caregivers (CCGs), during household visits as part of ward-based outreach teams. Patients who screen positive are required to produce a sputum sample of around 5mL of sputum, which is sent to a hospital-based laboratory for GeneXpert testing which shows whether or not TB is present and indicates rifampicin resistance. Production of a good quality sample is notoriously difficult; vigorous coughing is needed to produce sufficient sputum rather than saliva. Early morning samples have been preferred, leading to many patients being asked to take bottles home for collection. Provision for diagnosis using a chest-x-ray, cultures and LPA (Line Probe Assays) by a doctor is made for patients unable to produce sputum or for smear-negative patients with persistent symptoms.

Treatment must be provided by the public sector and is a six months’ short course, with a two-month intensive phase comprising four medications (rifampicin, isoniazid, pyrazinamide, ethambutol) provided as a fixed dose combination (RHZE), and four months continuation phase using rifampicin and isoniazid. Sputa are collected at pre-defined intervals to determine whether the intensive phase needs to be extended to three months and to classify treatment outcome. Until 2016, TB Treatment was provided to all TB patients using the standard DOTS (Directly observed treatment short course) (1). Since 2016, although DOTs is still recommended for specific groups who are at increased risk of treatment interruption, transfer of patients from the verticalised TB service to the Integrated Chronic Services Model is encouraged after two weeks, once the patient is deemed non-infectious (1, 2).

Treatment of MDR-TB changed dramatically in 2018 from prolonged (two years) therapy comprising at least one year of daily streptomycin to shorter injection-free treatment with bedaquiline (3). Although not officially a requirement, MDR patients are often admitted for initiation of treatment. Those with drug-sensitive TB may be admitted if clinically warranted.

| **Box 1: Relevant local, national and international guidelines in use** | |
| --- | --- |
| Ideal Clinic Policy (4)  Practical Approach to Care Kit (PACK) guidelines (5)  Adult Primary Care (APC) Guidelines (6)  National Tuberculosis Management Guidelines (1)  National Infection Prevention Control Guideline for TB, MDR-TB and XDR-TB (7) | Promotes integrated clinical services for all patients with a view that patients receive all care by one clinician.  PACK guidelines are clinical decision support tools, providing an evidence-based, comprehensive clinical approach to support the treatment of common symptoms, syndromes, diagnoses and conditions. The guides are designed for use in each consultation and starts with screening and a symptom-based approach, guides the diagnosis of common conditions, including priority chronic conditions and facilitates the routine care of the patient with one or several chronic conditions. PACK guides include PACK Child, Adolescent, Adult, Community and Home, thereby covering the life course and supporting all health workers in the primary care team.  APC guidelines are a comprehensive clinical tool for primary care of adults 18 years or older. The guidelines were developed using approved clinical policies and guidelines issued by the National Department of Health and is intended for use by health care practitioners. APC is being implemented as part of the Integrated Clinical services Management, a key focus within the Ideal Clinic.  The National Tuberculosis Management Guidelines provide South African department of Health’s guidance for management of TB, guidance on the management of adverse drug events and anti-retroviral initiation for patients co-infected with HIV.  The National Infection Prevention Control guidelines for TB, MDR-TB and XDR-TB provide guidance for staff to minimise the risk of TB transmission in health settings. Infection control measures should be established to reduce risk of TB transmission to both the general population and health care personnel. |

1. National Department of Health. National Tuberculosis Management Guidelines. Republic of South Africa; 2014.

2. National Department of Health. Adherence guidelines for HIV, TB and NCDs Republic of South Africa; 2016.

3. National Department of Health. Management of Rifampicin resistant Tuberculosis: A Clinical Reference Guide. South African Department of Health; 2019.

4. Ideal Clinic South Africa. Ideal Clinic Manual Version 16. Pretoria: National Department of Health, South Africa; 2015.

5. Cornick R, Picken S, Wattrus C, Awotiwon A, Carkeek E, Hannington J, et al. The Practical Approach to Care Kit (PACK) guide: developing a clinical decision support tool to simplify, standardise and strengthen primary healthcare delivery. BMJ Glob Health. 2018;3(Suppl 5):e000962.

6. Yau M, Timmerman V, Zwarenstein M, Mayers P, Cornick RV, Bateman E, et al. e-PC101: an electronic clinical decision support tool developed in South Africa for primary care in low-income and middle-income countries. BMJ Glob Health. 2018;3(Suppl 5):e001093.

7. National Department of Health. National Infection Prevention Control Guideline for TB, MDR-TB and XDR-TB. Republic of South Africa; 2015.
