# Supplementary material for: Identifying contextual determinants of problems in tuberculosis care provision in South Africa: a theory-generating case study
Source: Infect Dis Poverty. 2021 May 10;10:67. doi: 10.1186/s40249-021-00840-5 (PMC8108019; doi:10.1186/s40249-021-00840-5)
Supplement: Supplementary file 2 — Additional file 2. Guide for conducting observations of non-clinical areas. [file 40249_2021_840_MOESM2_ESM.docx]

**Promoting Person-Centred TB Care: A Mixed Method Study**

**Observations of Non-Clinical Areas Guide**

- What is the layout of the clinic for TB care provision? How is the area you are observing designed for TB patients?
- Which staff are working in the TB care areas? Numbers and different types.
- How are patients accessing care for TB?
- What gatekeepers are there? Reception staff? What conversations are patients having with staff in order to access care for their TB management ?
- What infection control procedures or protocols being followed and what is being implemented?
- How are patients with TB managed when they arrive as newly diagnosed or follow ups?
- Can you track how individual patients are screened, managed and treated? Are they having just one consultation or several?
- Note down any interesting quotes, particularly any points of tension between staff/patients.
