# Supplementary material for: Identifying contextual determinants of problems in tuberculosis care provision in South Africa: a theory-generating case study
Source: Infect Dis Poverty. 2021 May 10;10:67. doi: 10.1186/s40249-021-00840-5 (PMC8108019; doi:10.1186/s40249-021-00840-5)
Supplement: Supplementary file 3 — Additional file 3. Contextual domains of tuberculosis care provision. [file 40249_2021_840_MOESM3_ESM.docx]

**Table 3: Contextual Domains for Tuberculosis care**

| **Contextual Domain** | **CICI Description** | **Contextual Feature** | **Excerpts** |
| --- | --- | --- | --- |
|  | Distribution of disease/conditions, the attributable burden of disease as well as determinants of needs in human populations. Therefore, it also includes demographics. | *Macro (National) level:*  **HIV and antiretroviral treatment (ART).** HIV decreases immunity to TB infection and its prevalence largely determines TB incidence. ART increases immunity, protecting people with HIV from being infected with TB and then infecting others. ART coverage has increased since 2004 and TB incidence has consequently decreased since 2007 (1). |  |
| **Epidemiological context** |  | *Meso (District/Facility/Hospital) level:*  **High TB case fatality rates.** More than 11% for past four consecutive years. In 2017/8, 93% of those who died were registered as new cases (2). 79% died within the first three months of starting treatment. 75% died of pulmonary TB (2). Between 2014–2018, 71% of TB deaths were patients co-infected with HIV, 85% of whom were enrolled on anti-retroviral treatment (3). | ‘*We are having a high death rate, even with our drug sensitive TB there is a high death rate, but it also depends if the patient has been through a facility and missed out and also if you are having a high loss to follow up on the ARV* [Antiretroviral] *Programme you are going to see that type of issue*.’ (TB Manager) |
|  |  | *Micro (Individual) level:*  **HIV and TB co-infection.** Many clinicians reported that TB patients are also co-infected with HIV. 71% of TB deaths were co-infected with HIV and has been started on anti-retroviral treatment (3). | *We have at least close to 65% of our patients are co-infected so we have to make sure that if they are on the TB programme that they need to be on ARVs also.’ (TB Manager)* |
| **Socio-economic context** | Economic resources of a community and the access of a population to these resources. It also shows the relationship between an economy and its society. | *Macro (National and International) level:*  **Unemployment and poverty.** Many TB patients come from households with low socio-economic status, where unemployment is rife and living conditions are sub-optimal which results in a high TB burden. | ‘O*ur patients most of them are not working. Sometimes they will report that they don’t have food at home.’* (Facility Manager) |
|  |  | *Meso-level:*  **Electricity load-shedding.** Due to national electricity shortages, load shedding affects laboratories systems and therefore delays release of results and initiation of treatment. | *‘Just an example from today, we don’t have any blood results, or sputum results.  So, there are quite a few sick patients that I took bloods on yesterday, and when I checked on the system today there are no results. I called the lab, they said there is load shedding, the system is down. So, I mean, if there’s any correctable factors, I can’t really do it. Patients I need to start ARVs, I need results for. I can’t do it, I don’t have any results.*’ (Doctor) |
|  |  | *Micro-level:*  **Access to facility.** Patients described being unable to adhere to their TB treatment because of a lack of finances to travel to the facility.  **Poor Nutrition**. Many patients report not having sufficient food/nutrition to take their treatment. | ‘*They start their treatment and promise they will be able to come whatever but eventually when they can’t afford the taxi fees or the bus fare, that is when they start defaulting.*’ (Facility Manager)  *‘I mean generally, we see quite a few patients coming in in extremely poor condition; malnourished in respiratory distress, oxygen dependent.’* (Doctor) |
| **Political context** | The distribution of power, assets and interests within a population, as well as the range of organisations involved, their interests and the formal and informal rules that govern interactions between them. The domain also comprises the health care system and the securing of its accessibility. | *Macro-level:*  **The WHO End TB Strategy:**  The strategy aims to end the global TB epidemic, with targets to reduce TB deaths by 95% and to cut new cases by 90% between 2015 and 2035, and to ensure that no family is burdened with catastrophic expenses due to TB (4).  **(**<https://www.who.int/tb/post2015_strategy/en/>)  **90-90-90 targets:**  Mimicking the UNAIDS 90-90-90 targets, this is to screen 90% of all people with TB, as part of this approach to reach 90% of key populations (5) and to achieve at least 90% treatment success for all people diagnosed with TB (6).  **Ideal Clinic Policy:**  The Ideal Clinic policy encourages integrated clinical services for all patients including those with TB. Ideally a patient can receive all care by one clinician instead of multiple clinicians for e.g. TB treatment, ARV treatment administered by one clinician (7). | *‘So, we are looking at 90-90-90. Screening 90% of patients and above, making sure 90% are initiated and 90% are sustained and compete their treatment, but we are far from that I think*.’ (TB District Manager) |
|  |  | *Meso/micro-level:*  **Continued verticalized care**: Despite the implementation of Ideal Clinic policy, different clinicians are allocated to see patients with TB and HIV separately. They are considered specialists compared to other nurses who report lacking knowledge to manage TB patients. | ‘*My challenge is here if there was someone doing the HIV programme they will just concentrate on the ARV programme, the person that is doing the TB programme will have to concentrate on that. In the Ideal Clinic situation if you walk into this stream, you will get everything done. But it doesn’t work that way, I mean I have complaints from the TB co-ordinators in the facility that if they are not there, the patients are not seen to*.’ (TB Manager) |
| **Institutional context (Added to CICI domains)** | Workforce arrangements, staff training, organisational rituals and patient flows. | Meso/micro-level:  **Biomedical focus of care.** Patients and clinicians described that care for TB patients was primarily focused on the clinical aspect of ensuring the patients were adherent to treatment.  **Ill-prepared for treatment side effects.** Patients seemed ill prepared for the side effects they were experiencing and how to manage these. | *‘I didn’t talk to any of the doctors or nurses who were treating me but also nobody ever asked me how I am feeling, how I am coping. The clinic focuses more on the biological. And not that they ask how treatment is going, their focus is the drugs and the drugs got in and you didn’t vomit but they never actually ask how I was doing on treatment or how I was feeling in the process, so for me I feel like it was completely ignored.’(TB Proof member)*  ‘*We give them health education on how to take after they've taken treatment, they need to tick saying I’ve taken treatment today, and if they have not taken treatment, also tell them to indicate that they did not take treatment on the day. So, at the end of the course of treatment then we go back to cover for days they did not take treatment. Also we just give them support, like taking treatment as expected.’* (Nurse)  *Interviewee: Yes, they did tell me that there are changes that that I am going to experience but they didn’t tell me specifically what are it that I am going to experience.’*(Patient) |
|  |  | Meso level:  **Patient flow.** Where previously TB patients were seen in ‘park homes’ (temporary structures used due to lack of formal infrastructure to accommodate patients outside the main clinic), with the implementation of Ideal Clinic they need to receive services within the clinic. Although this reduces stigmatising/discrimination it exposes other patients to TB as the TB room is positioned near consult rooms for patients with chronic illness and children. TB patients include those known to have TB and already on treatment, and those yet to commence treatment. Those not on treatment pose a greater risk of transmission.  **Disorganised and unclear referral pathways.** Numerous cases were reported where patients presented repeatedly at clinics and were not screened properly with diagnosis occurring months later in hospitals. Poor communication and lack of information continuity across services contributed to this situation resulting in delayed initiation of treatment.  **Poor quality sputum collection.** High sputum rejection rates; commonly insufficient specimens (40%) and leakage (34%) (January to August 2019).  **Inconsistent TB screening.** Quality of screening varied within and across facilities. Screening checklists inconsistently used, not all questions asked and questions asked from memory.  **Medication shortages and TB/ART coordination.** Doctors reported not having required medication and needing to prescribe contraindicated substitute | ‘*It is a problem we are facing, because of the ideal, the streams, the Ideal says we must have three streams. All the time we used to take TB and do them in the park homes because there is enough ventilation there. So now they said those patients must come this side to be seen, because they said if we separate them we are discriminating the patients. Of which we are seeing it is a problem on our side because we were worrying because we have small babies, they are also sitting on the same area with these people, because there is no enough ventilation*.’ (Facility Manager)  *‘I recently had an MDR case we were trying to follow who was diagnosed right in this clinic. The sister made arrangement for the patient to go to the hospital to be initiated, she got an ambulance and the patient was taken to hospital, was lost into the system in the OPD* [Outpatient Department]*, given treatment and sent back home. The patient was unwell and came back again and was sent to gate clinic now as she didn’t come in an ambulance. They suspected her having TB, they took a GeneXpert and I don’t know for some reason it came back negative but clinically they looked at her and they started her on TB treatment. She came back again not feeling well, so now they decide to send her to the TB doctor and then it was picked up, this was MDR patient. So, she was started on MDR treatment, so that was like about three weeks after.’ (TB Manager)*  *‘Sometimes the lab will tell you the sputa is insufficient; the sputum is a poor quality. The results sometimes you find the sister is so busy, she can’t even follow those patients because they have to repeat the sputa if the results came poor quality. I think we can conquer TB by that, if we call all those with poor quality to cough again*.’ (Facility Manager).  ‘*We do have documents that we use but we don’t carry them daily our document is our minds now.*’ (Community caregiver)  *‘Last year there was a shortage of, I think it was moxifloxacin* [used for treatment of MDR-TB]*, I think. So, we had to substitute a drug, basically which we can't really do. ARV wise, there is certain ARVs we can't give with the MDR treatment, because we need to substitute it, but then those drugs are out of stock. Then you basically need to switch someone to a different ARV regimen where that would actually be very detrimental to their health. Either because of contraindications such as anaemia. So, you need to get an anaemic patient medication that actually worsens anaemia, or because it can actually cause resistance of the HIV virus. So, this happens with the TB treatment, with the ARVs, with other medications. Potassium supplementation is out of stock at the moment.’* (Doctor) |
| **Socio-cultural context** | Explicit and implicit behaviour patterns, including their embodiment in symbols and artefacts; the essential core of culture consists of historically derived and selected ideas and values that are shared among members of a group. It not only refers to the conditions in which people are born, grow, live, work and age but also embraces the social roles a human being takes on as a family member, community member or citizen and the relationships inherent to these roles. Constructs such as knowledge, beliefs, conceptions, customs, institutions and any other capabilities and habits acquired by a group are covered by this domain. | *Micro-level:*  **Providing incorrect addresses.** Facility staff reported that patients often provide incorrect addresses to the clinic, making it difficult for them to be followed up. Reasons stated were that patients preferred certain clinics and lied in order to receive treatment in their selected clinic. Sometimes the reason for selection is proximity to work and stigma (fear of being seen by neighbours in the local clinic)  **Preference for doctors.** Clinicians report that patients often prefer to be seen by doctors and therefore community centres or hospitals are flooded with patients and most diagnosis occur at these levels.  **Traditional healers.** Patients commonly use traditional healers, who may in some cases be the first point of care before being screened at the clinic. Often this was reported as being significantly influenced by family members. According to several staff, patients often hide their use of traditional healers from clinicians.  **Delayed care seeking**. Clinicians described patients being afraid of diagnosis, with a tendency to present or take up referrals very late, sometimes even a month or two after providing sputum sample.  **Self-isolation**. Some patients reported that they self-isolated due to fear of infecting others.  **Loss of sense of self**. Patients frequently reported how key aspects of their lives had changed since diagnosis and commencing TB treatment. In each case, something had been lost, including:   - Control of the body - exhaustion, vomiting, joint pains, appetite, weight, bowel and bladder function, body odour. - Mental state – *‘I just couldn’t control my actions, I felt like I was losing my mind.’* - Professional role and income - Social contact - Personal relationships and intimacy - Independence, burdening others | *‘Patients are defaulting their treatment. They do default, they give wrong addresses, even if you stress it is important to give the right address, but they do give the wrong address.*  *Do you know why they give us wrong addresses is because they like to come to this clinic, they don’t like to go to their respective clinics. It is because they know if they give the right address you are going to treat them and refer them to the nearest clinic. So, they like this clinic. That is why they give the wrong addresses.’* (Facility manager)  *‘The other challenge is we have a CHC* [Community Health Centre] *in* [name of hospital] *and then we have* [name] *hospital, patients like to be seen by a doctor, so they will flood these areas. If you look at* [name] *hospital, if you look at they are diagnosing 350 patients per quarter, you find in* [name of hospital] *which has a population of about 37000 that are diagnosing 150 patients, so they are flocking in to be seen by a doctor. The Community health centre has a doctor there, so they are flocking in to be seen, further if they want to start treatment there.’ (*TB Manager)  *‘Usually we ask, if you are really not sure what's happening. If someone is really the case is confusing you, you can't really figure out what's happening. They'll say no, and what I usually do is I'll ask the nurses to go and ask, because they are too scared to tell the doctor usually. They don't really willingly disclose herbal medication. If I can guess, it's because they scared you're going to be angry with them, I guess or instruct them to stop taking it. I think, which is what any doctor will tell you, please stop taking the herbal medication. They have quite strong faith in it*.’ (Doctor)  ‘*People present very late, because they start getting sick and basically, they’re told by the community, it's a very common thing that if you come to hospital you're going to die. So, hospital is almost a last resort. I work in casualty as well, after hours so I see that quite a lot. I mean people present extremely late. It's really within hours of dying*.’ (Doctor)  ‘*I usually sit alone because I do not want to spread TB to them.’* (Patient)  *‘It disrupted every part of my life’* (TB Proof representative, treated in 2012) |
| **Ethical context** | Reflections of morality, which encompasses beliefs, standards of conduct and principles that guide the behaviour of individuals and institutions. | *Meso-level:*  **Hospitalisation of infectious patients.** Clinicians reported that they would hospitalise patients until their Acid-Fast Bacilli (AFB - test for TB bacteria) was negative for two consecutive months, indicating an ethical practice of protecting the wider community from infection. (NB was the standard test for TB before GeneXpert was introduced. It is still used widely to refer to a TB sputum test. AFBs are no longer completed, bur rather have been replaced by GeneXpert and LPA (Line-Probe Assay).  Meso-level:  **Difficulty implementing infection control measures**. Despite the need to implement infection control measures at facilities, clinicians report that these are not always practised.  Micro level:  **Stigmatisation, stress and fear of TB amongst staff.** Clinicians and managers expressed fear of TB and reluctance to be responsible for providing TB care. TB care in facilities was frequently seen as the sole responsibility of one nurse, leaving little support if the nurse was to contract TB symptoms.  **Stigmatising experiences for patients.** Some patients reported no stigma from relatives whilst others reported some stigma from friends seen through reduced interactions.  Staff and patients emphasised that many patients with HIV also have TB, indicating the potential for a dual stigma. | *‘We admit, or we try and admit all patients, just to sort out all social issues and then within about a week we have the sputum results. So basically, there is a smear that says AFB either negative, which then tells us there’s a low possibility of the patient actually be infectious to the community or relatives, and then we can think about discharging and treating as an outpatient, if the patient’s condition permits it. Obviously if they’re ill and they still need further inpatient treatment, we keep them here and discharge once they've stabilized. Otherwise anyone with AFB positive stays here until they have two negative AFBs, basically two months in a row.’* (Doctor working in MDR ward)  *‘If you say wear enough masks you know each and every person will wear a mask. But they are not wearing them in the consultation. They will have it kept there but you will find it is just not worn.’* (TB Manager)  ‘*It is difficult with TB because we are afraid, TB is a communicable disease. Sometimes I am afraid because I can get it. I am stress, I am stressed, If I can get it from working with patients. Even when I wake up in the morning, I am stressed. Why I am stressed because I have got in my house I have got my child, I have got two chronic, I have got my child who has diabetes and it is easier to get TB and my husband is also a chronic disease, so I am afraid if I am getting TB who is going to helping me, because me I am helping those patient.’* (TB nurse)  ‘*My friends from school found out from me or they heard me speaking at medical school about it. So I did feel stigmatised by them, because a lot of them just decreased their interactions with me. I heard rumours from others that maybe I had HIV and it really hurt*.’ (TB Survivor)  ‘*They said HIV and TB are “best friends*”!’ (Patient) |
| **Legal context** | Rules and regulations that have been established to protect a population‘s rights and societal interests. | *Macro level:*  **Occupational Health and Safety:** The Occupational Health and Safety Act of 1993, Act No 85 of 1993 [Section 8] states that employers have a duty to their employees to “provide and maintain, as far as is reasonably practicable, a working environment that is safe and without risk to the health of his employees.” (8). The Act does not however include regulations specific to TB or health care.  *Meso level:*  **Bureaucracy.** Tensions in lines of reporting between programme management and line management of clinical services. Clinics report to both, but their primary allegiance is to clinical services who are their direct managers and responsible for managing their performance.  Patients seen privately need to be referred back to public health facilities for treatment. | ‘*You know we can’t really go to a facility if we see something wrong, we have to report it to the operational manager. And we can’t go and discipline that person. We probably take a report to the district office and then nothing really. I mean we are sitting with many challenges that are known about and there is nothing actually happening*.’ (TB District Manager) |
| **Geographical context** | Broader physical environment, landscapes and resources, both natural and transformed by humans, available at a given location, including infrastructure at a given location, which could result in geographical isolation. | *Meso:*  **Poor ventilation.** Clinic infrastructure limited ability to provide good ventilation with crowded corridors as waiting areas**.**  *Micro:*  **Employment demands**. Some patients are lost to follow up as they often come ‘home’ for treatment and then move back to the cities where they work once they feel better without obtaining a transfer letter to complete treatment.  Manufacturing industries provide limited support for patients to attend scheduled appointments. Absence of communication between clinic and employer limits tracing, TB treatment initiation and completion. | *‘There is not enough ventilation, that is why we become sick now and then, sick now and then. Because there is no ventilation in this clinic.’* (Facility manager)  ‘*That is the other problem if they very ill they'll come home from Johannesburg, for instance They'll come home they’ll be brought here, we’ll do everything, we’ll initiate the treatment. The next thing they supposed to come back. We trace them even telephonically, ‘oh no I'm in Johannesburg. I've gone back to work.’* (Facility Manager)  *‘They don’t get paid. I once had my husband’s relative she was positive. So the sister told me, okay, could you please make sure so and so come. I also rang her to say she should come. So as I rang her, you know the answer she told me, I’ll come on such and such a date. And I say no, why? Because you need to come by tomorrow. I rang her in the afternoon, you have to come tomorrow. Oh no, I have to go to work, you know that nobody is working here. So I don’t know what is happening, how many people are having TB really in the industries because its so difficult.*’ |

1. World Health Organisation. Tuberculosis profile: South Africa 2021. Available from: <https://worldhealthorg.shinyapps.io/tb_profiles/?_inputs_&entity_type=%22country%22&lan=%22EN%22&iso2=%22ZA%22>.

2. Hlongwa M, Ngozo J, Tshabala M. Profiling TB deaths in Amajuba District, KwaZulu-Natal province, South Africa2020.

3. Hlongwa M, Ngozo J, Tshabalala M. Characteristics of patients who died while on TB treatment in Amajuba District 2017-18. Durban: Department of Health; 2019.

4. World Health Organisation. The End TB Strategy: Global strategy and targets for tuberculosis prevention, care and control after 2015. WHO; 2014.

5. Friedland BA, Sprague L, Nyblade L, Baral SD, Pulerwitz J, Gottert A, et al. Measuring intersecting stigma among key populations living with HIV: implementing the people living with HIV Stigma Index 2.0. J Int AIDS Soc. 2018;21 Suppl 5:e25131.

6. Republic of South Africa. Let Our Actions Count: South Africa's National Strategic Plan for HIV, TB and STIs: 2017-2022. Pretoria: South Africa; 2017.

7. Ideal Clinic South Africa. Ideal Clinic Manual Version 16. Pretoria: National Department of Health, South Africa; 2015.

8. South African National Department of Health. National Infection Prevention and Control Strategic Framework 2020.: <https://www.nicd.ac.za/diseases-a-z-index/covid-19/covid-19-guidelines/national-infection-prevention-and-control-strategic-framework/>; 2020.
