# Supplementary material for: Identifying contextual determinants of problems in tuberculosis care provision in South Africa: a theory-generating case study
Source: Infect Dis Poverty. 2021 May 10;10:67. doi: 10.1186/s40249-021-00840-5 (PMC8108019; doi:10.1186/s40249-021-00840-5)
Supplement: Supplementary file 4 — Additional file 4. Contextual determinants, hypothetical propositions and intervention components for tackling problems in TB care. [file 40249_2021_840_MOESM4_ESM.docx]

**Table 4 – Contextual determinants, hypothetical propositions and intervention components for tackling problems in TB care.**

| **Problem** | **Contextual Domain: Level** | **Contextual Determinants** | **Evidence** | **Hypothetical Propositions** | **Recommendations for intervention components and implementation strategies** |
| --- | --- | --- | --- | --- | --- |
| **Delayed diagnosis at community and facility level** | Epidemiological; institutional; political; socio-cultural: Macro, meso and micro | - Verticalised TB provision within facilities.  - Inadequate screening of patients at community and facility level.  - Inefficient referral pathways (private/clinic/hospital)  -Lack of patient knowledge of how to provide sputum sample.  - Poor documentation of TB presumptive patients.  - Delays in test results being received by clinics or disclosed to patients.  - Stigmatisation of TB shaping loss to follow-up, incorrect addresses, fear of diagnosis and treatment. | - One nurse dedicated to TB care. Reports that TB not managed by other clinicians.  **-** Observations and patient reports identified inconsistent screening.  Some patients reported accessing multiple public and private services before diagnosis.  -Delayed diagnosis was reported due to poor sputum collection.  High levels of rejected samples cause delays and patients are required to return to clinic.  - Staff perceived patients being afraid of diagnosis and treatment, leading to delays in returning to clinic. | **Delayed diagnosis caused by interaction between:**  ***Macro:*** Cultural stigmatisation of TB; vertical disease programmes; fragmented public and private healthcare provision; limited resources to support efficient and timely screening, testing and diagnosis;  ***Meso:*** Inadequate screening of the community, poor sputum collection and test result turnaround, poor referral pathways and communication between contact points, community fears/stigmatisation of TB; and  ***Micro:*** poor collection of sputum by nurses resulting in rejected samples and therefore delayed diagnosis. | 1. Provide staff training to improve identification and management of patients. 2. Strengthen screening and case finding at community and clinic level. 3. Strengthen clinical practice and records of testing, diagnosis and treatment initiation at community and clinic level. 4. Improve sputum collection at facilities and educate patients about providing sputum samples. 5. Strengthen referral pathways within and between public and private sector. 6. Educate communities about TB, potential for full recovery, importance of screening, need for support from families. 7. Improve IT systems for data sharing between public and private sector. |
| **Patients lost to follow up after diagnosis or end of treatment initiation phase** | Socio-economic; institutional; geographical; socio-cultural: Macro, meso, micro | - Poverty restricting access to food leading to poor medication adherence and access to clinics. Patients working away from home leading to loss to follow-up and not completing treatment  - Limited preparation for treatment side-effects, focus on educating patients about treatment adherence.  - Clinics not able to contact patients leading to loss of follow-up and continuation of treatment | - Staff and patients reported limited ability to access clinic.  - Patients reported knowing very little about TB treatment side-effects and little support for psychosocial problems  - Staff reported patients providing incorrect addresses (to avoid going to clinics near home or home visit from CCG) | **Patients are lost to follow up due to interaction between:**  ***Macro:*** Cultural stigmatisation of TB, poverty, geographical distribution of employment opportunities, biomedical focus of care on treatment adherence;  ***Meso:*** little preparation and support for treatment side-effects, inaccurate records of patient addresses, discontinuity of information across services, and  ***Micro:*** poor access to nutrition and clinics, employment migration, avoidance of clinic. | 1. Improve collaboration between industry and health sectors to adhere to TB medication. 2. Increase financial support for TB patients to complete treatment phases. 3. Educate/counsel patients around treatment side-effects and support through to completion. 4. Strengthen resources to minimise medication stockouts. 5. Strengthen information sharing between clinics when patients move for work |
| **Limited support for psychosocial needs of staff and patients** | Institutional; socio-cultural: Macro, meso, micro | - WHO policy emphasises TB as an epidemic that needs to be controlled through treatment adherence with limited focus on the support of the patient on this journey.  - Verticalised TB care  - TB Stigma/Fear of infection  - Lack of training on TB management. | **-** Staff and patients reported the focus of TB care primarily on the clinical aspects of care and treatment adherence. Little support is offered psychologically or with management of side effects of the treatment.  - One nurse usually allocated to see TB patients. There is a reluctance amongst staff for this role because of fear of infection. | **Macro** policy and discourse of TB as an epidemic shapes how staff are trained and clinical orientation towards TB screening, diagnosis and treatment adherence at a **meso-level**. Having only one nurse allocated to TB creates a stigmatised culture and emotional burden for TB nurse, limits support for staff to manage TB, as well as increasing exposure for allocated nurse to risk of infection. Clinical orientation regulates the form and content of patient contacts at a **micro-level**, limiting discussion about emotional and psychological problems for patients after starting treatment and may also be determinants of patient’s non-adherence to treatment. | 1. Strengthen knowledge of TB management and risk of infection for all staff at clinics. 2. Develop TB policy to promote sharing responsibility of TB across all clinical staff. 3. Provide psychosocial support for TB nurse to manage demands of role and fears of infection. 4. Strengthen psychosocial support for TB patients during transition from intensive to continuation treatment. 5. Strengthen support of TB patients discharged from hospital to community settings. |
| **Limited integration of TB with HIV and other NCDs** | Institutional: Macro, meso, micro | - Verticalised TB care  - Medication shortages potentially leading to prescribing contraindications. | - Despite Ideal Clinic policy, staff reported inconsistent integration of TB, HIV and other NCDs. | Verticalised care and medication shortages limit effective TB/ART coordination | 1. Strengthen facilities capacity to integrate TB, HIV and chronic NCD care. 2. Provide staff training on managing multi-morbidities. 3. Promoting TB as ‘everybody’s business’ (1) |
| **Inadequate Infection Control alongside policy to minimise stigma** | Institutional; socio-cultural: Macro, meso, micro | - Poorly ventilated clinics  - Variable and less than adequate infection control measures within facilities.  - Wearing masks not routinized and normalised within clinics. | - Despite being aware of infection control, observations identified that the main measure was to open windows and doors.  Wearing of masks was mainly limited to TB consultation rooms. Some patients were observed wearing masks but often they were not properly fitted. | **Inadequate infection control caused by interaction between:**  **Macro:** Ideal clinic policy aimed at destigmatising TB requiring TB patients not to be isolated from non-TB patients;  **Meso:** Clinics implementing Ideal Clinic policy seating TB patients next to non-TB patients in waiting areas. Poorly designed clinics restricting ventilation; and  **Micro:** lack of routinized use of masks results in higher rates of transmission of TB between patients in the clinic and exposes more staff to the infection. | 1. Policy to clarify practice of infection control measures at clinic and community level, which do not also function to reinforce TB stigma. 2. Develop health promotion messages to educate and normalise infection control and minimise stigma. |
| **Limited access to care for patients with MDR-TB** | Institutional; ethical: Meso | . Management of MDR TB centralised at hospital  - PHC staff not equipped to manage MDR-TB | **-**According to clinicians, all MDR as well as some drug sensitive TB patients are hospitalised and initiated on treatment. | The centralisation of MDR-TB at hospitals limits access to treatment for patients with MDR-TB. This is a particular problem for those who are employed who cannot be hospitalised for extended periods.  Prolonged hospital admissions increase sense of isolation if far from home with no visitors. | 1. Strengthen support for MDR-TB patients discharged from hospital to community settings |

1. World Health Organisation. Everybody business: strengthening health systems to improve health outcomes: WHO’s framework

for action. Geneva, Switzerland: WHO; 2007.
